# Prevalence of HIV and other infections and correlates of needle and syringe sharing among people who inject drugs in Podgorica, Montenegro: a respondent-driven sampling survey

**DOI:** 10.1186/s12954-015-0039-0

**Published:** 2015-02-28

**Authors:** Dragan Lausevic, Senad Begic, Boban Mugosa, Natasa Terzic, Zoran Vratnica, Itana Labovic, Ivana Bozicevic

**Affiliations:** Institute for Public Health, Podgorica, Montenegro; HIV/AIDS Office, United Nations Development Programme, Podgorica, Montenegro; World Health Organization Collaborating Centre for HIV Surveillance, School of Medicine, University of Zagreb, Zagreb, Croatia

**Keywords:** People who inject drugs, HIV, Hepatitis C, Survey, Montenegro

## Abstract

**Background:**

People who inject drugs (PWID) have significantly higher rates of blood borne and sexually transmitted infections due to unsafe injecting practices and risky sexual behaviors.

**Methods:**

We carried out an HIV bio-behavioral survey using respondent-driven sampling (RDS) in people who use drugs (PWID) in Podgorica, Montenegro in 2013 in order to determine the prevalence of HIV, hepatitis C (HCV), hepatitis B surface antigen (HBsAg) and risk behaviors. Data were analyzed using RDS Analyst and SPSS 12.0 to obtain prevalence estimates of key bio-behavioral indicators and assess correlates of needle and syringe sharing using multivariate logistic regression.

**Results:**

A total of 402 PWID were recruited. HIV prevalence was 1.1%, while the prevalence of HCV and HBsAg was 53.0% and 1.4%, respectively. In the multivariate analysis, significant correlates of needle and syringe sharing in the past month were being older than 26 years, female, injecting drugs more than once per day, injecting in parks or on streets, not being able to obtaining free-of-charge sterile needles and syringes and reporting more than four partners in the past 12 months.

**Conclusions:**

The results indicate that the HIV epidemic in PWID in Montenegro might still be at a low level, though the HCV epidemic is well-established.

## Background

People who inject drugs (PWID) have significantly higher rates of blood borne and sexually transmitted infections (STIs) due to unsafe injecting practices and risky sexual behaviors [1]. Unsafe sexual behaviors facilitate transmission of STIs to their sexual partners, thus increasing the risk of the spread of HIV and other STIs [2].

Montenegro, a former Yugoslav republic, is a small, newly independent country in southeastern Europe with a population of 620,000 people and a territory of around 13,000 km^2^. Podgorica, the capital of Montenegro, is the largest city with around 200,000 inhabitants, constituting almost a third of a total population of Montenegro [3]. Podgorica is situated almost in the geographical center of the country.

Since the beginning of HIV case reporting in 1989 until the end of 2013, 153 HIV cases were reported of whom five (2%) were reported as being due to injecting drug use, 42% as homosexual transmission and 44% as heterosexual transmission [4]. In the period 2005–2013, the number of newly reported cases of HIV ranged from 1.1 to 2.2 per 100,000 population.

HIV prevention interventions and harm reduction programs in PWID have been available in Montenegro since 2004 [5]. Currently, there are two drop-in centers for PWID in Podgorica established in 2010 and one center for Voluntary and Confidential HIV Counseling and Testing (VCCT) established in 2003. The services of the Center for Methadone Substitution Therapy have been available since 2005 while those of the Center for Rehabilitation of Drug Addicts since 2008. Drop-in centers are managed by two non-governmental organizations (NGOs) while the VCCT is operated by the Institute for Public Health (IPH). PWID can obtain sterile injecting equipment free-of-charge at drop-in centers and via outreach services, as well as at the premises of the Primary Health Care Center in Podgorica. The Global Fund to Fight AIDS, Tuberculosis and Malaria has been a major funding source for HIV prevention among key populations in Montenegro since 2006.

Integrated bio-behavioral surveys (IBBS) using respondent-driven sampling (RDS) were implemented in Podgorica in 2008 and 2011 and found an HIV prevalence of 0.4% in 2008 and 0.3% in 2011, while the prevalence of hepatitis C (HCV) was 53.6% and 55.0%, respectively [6,7].

The aim of this paper is to present the results of the IBBS carried out in PWID in Podgorica in 2013, specifically the prevalence of HIV, HCV, and hepatitis B virus surface antigen (HBsAg), and behaviors relevant for transmission of HIV and HCV, as well as correlates of sharing of needles and syringes for drug injection.

## Methods

### Sampling

We conducted a cross-sectional bio-behavioral survey among PWID in Podgorica using RDS [8,9]. Recruitment was initiated with five *seeds* (initial participants), of whom four were males and one female. Seeds were diverse in respect to age and a place of residence in the city. Seeds, as well as each participant who completed the survey, were provided with three coupons to be used in recruiting maximum of three eligible participants. Eligible participants were individuals older than 18 years living in Podgorica for at least 3 months during 12 months before the survey and who injected drugs for non-medical purposes in a month preceding the survey. Before agreeing to participate in the survey, participants were described the nature and characteristics of the survey and were asked for a verbal consent to participate. Following the consent, participants completed a questionnaire and were afterwards provided with pre-test HIV counseling and taken a sample of venous blood. Primary monetary incentive and three coupons for peer recruitment were given to participants after completion of the abovementioned steps of the survey. Participants were instructed to re-visit the survey site in 14 days in order to collect recruitment-related secondary incentives and test results. Post-test counseling was provided after issuing HIV, HCV, and HBsAg test results. Data collection lasted from November to December 2013.

A sample size calculation was designed to detect a 9% decline in needle and syringe sharing in the past month with 80% power and an alpha error of 5%, from a value of 13.6% in the survey carried out in 2011. It was estimated that the sample size of 376 PWID was needed and that was rounded up to 400 participants.

### Behavioral questionnaire

We used the same behavioral questionnaire as in the 2011 survey, with minor modifications. The questionnaire was based on a standardized behavioral questionnaire for PWID published by the Family Health International and was slightly modified and adapted to the country context [10]. It sought data on socio-demographic status, knowledge regarding the modes of HIV transmission, patterns of sexual and drug using behaviors, past HIV testing, and awareness and utilization of harm reduction services. A questionnaire was self-administered. In case that a participant needed help during completion of a questionnaire, trained study staff provided appropriate explanations and assistance. Data were also collected on participants’ social network sizes that were assessed as the number of PWID they know by name, are older than 18 years, who lived in Podgorica, and have seen them in the past 3 months.

### Laboratory methods

Blood samples were tested in the laboratory of the Center for Medical Microbiology of the IPH, which serves as a national referral center for HIV testing. Serum samples were tested for the presence of HIV-1/2 antibodies and p24 antigen using ELISA (HIV Ab&Ag, Dia.Pro Diagnostic Bioprobes srl., Milano, Italy), while confirmatory HIV-1/2 testing was done by NEW LAV BLOT I and NEW LAV BLOT II (Bio-Rad, Marnes-la-Coquette, France). ELISA was also used for detection of antibodies against HCV (HCV Ab, Dia.Pro Diagnostic Bioprobes srl., Milano, Italy) and HBsAg (HBsAgone Version ULTRA, Dia.Pro Diagnostic Bioprobes srl., Milano, Italy).

All survey procedures were conducted by trained personnel. Ethical approval was granted by the Ethical Committee of the IPH in Podgorica.

### Analysis

Univariate analysis to calculate key socio-demographic, behavioral, and biological indicators was done using Respondent-Driven Sampling Analyst (RDS-A) statistical software that provides weighted population estimates with 95% confidence intervals (95% CI) of the variables of interests [11]. Bivariate and multivariate logistic regression analysis was done using SPSS 12 statistical software package on unweighted data (SPSS for Windows, Version 12.0). Data on seeds were excluded from the analysis, as per RDS analysis procedures.

We assessed factors associated with needle and syringe sharing in a month before participation in the survey using logistic regression analyses. Sharing needles and syringes was defined by either giving needles and syringes that a respondent used by himself/herself to other PWID or using needles and syringes for injecting drugs that were already used by other PWID. The following variables were considered potential correlates of needle and syringe sharing: age, gender, monthly income, frequency of injections in the past month, number of partners in the last 12 months, injecting drugs in an outdoors setting (in parks or on streets) during past month, and being given free-of-charge needles and syringes by NGOs and the Primary Health Care Centre in the past 12 months. Results are presented as odds ratios (ORs) with 95% CI. Age and gender as confounders and variables associated with sharing needles and syringes at the level of *p* < 0.05 in the bivariate analysis were included in the multivariate logistic regression model. The cutoff for considering a result to be statistically significant in the multivariate analysis was set up at *p* < 0.05.

Missing values were excluded from the analyses.

## Results

### Recruitment patterns, socio-demographic characteristics, and knowledge of HIV

Five seeds recruited a total of 402 eligible participants. The median social network size was six (interquartile range (IQR) = 3–10). The mean number of waves was 8.2, ranging from 4 to 10. We distributed a total of 1,202 coupons.

A majority of survey participants were men (90.1%). The median age of respondents was 32 (IQR 28–35) years. A majority of PWID completed either secondary (60.5%) or primary (23.5%) school. Being unemployed was reported by 58.2% of respondents, while 64.7% reported an income of <150 EUR per month.

A majority of respondents knew that it is possible to decrease the risk of HIV transmission by not sharing needles and other injecting equipment (86.1%) and by proper and consistent use of condoms during sexual intercourse (85.4%), but only 37.1% had comprehensive knowledge of HIV^a^.

### HIV testing and HIV, HCV, and HBsAg prevalence

A majority of PWID (75.6%) knew where it was possible to be tested for HIV.

Never been tested for HIV was reported by 57.7% of PWID while 18.9% were tested during the last 12 months.

HIV prevalence was 1.1%, while the prevalence of HCV and HBsAg was 53.0% and 1.4%, respectively. HCV prevalence was 31.5% in those younger than 25 years of age and 57.4% among older than 26 years.

### Sexual risk behaviors

A majority of PWID (70.1%) were sexually active during a month preceding the survey. A half of respondents reported having regular partners at the time of the survey, and 24.2% used condom at last intercourse with a regular partner (Table 1). Before having the first sexual intercourse with a current or last regular partner, only 28.6% of PWID discussed HIV status with that partner. Having non-regular partners during the past 12 months was reported by 62.2% of PWID, and 46.4% of these reported consistent condom use with this type of partners.

**Table 1:** **Sexual and injecting drug use behaviors in people who inject drugs in Podgorica, Montenegro, 2013**

	***n*** **/** ***N***	**Sample prevalence %** ^**a**^	**RDS** ^**b**^ **prevalence estimates % (95% CI)** ^**c**^
Currently has regular sexual partner	217/401	54.1	50.9 (44.1–57.6)
Used condom during last sex with regular partner^d^	100/396	25.3	24.2 (19.1–29.2)
Discussed HIV status before having first sexual intercourse with regular partner^d^	127/401	31.7	28.6 (22.8–34.3)
Had non-regular sexual partner in the past 12 months	251/399	62.9	62.2 (56.2–68.4)
Used condoms consistently during sexual intercourse with non-regular partners in the past 12 months	109/251	43.4	46.4 (37.1–55.8)
Total number of sex partners in the past 12 months			
0–1	123/392	31.4	31.1 (25.3–36.8)
2–3	121/392	30.9	34.0 (27.2–40.8)
4–5	63/392	16.1	16.7 (11.8–21.5)
6–10	63/392	16.1	13.7 (9.6–17.8)
≥11	22/392	5.6	4.5 (2.3–6.8)
Ever had sexual intercourse for which received or gave money or drugs	216/400	54.0	48.8 (42.0–55.6)
Used condom during last sexual intercourse for which gave or received money or drugs	169/216	78.2	79.5 (71.6–87.5)
Used drugs before sexual intercourse in the past 12 months			
Never	16/398	4.0	3.5 (1.4–5.5)
Rarely	30/398	7.5	11.9 (7.4–16.4)
Sometimes	83/398	20.9	22.4 (17.0–27.8)
Often	269/398	62.2	62.2 (55.9–68.4)
Type of drugs used in the past month			
Heroin	381/401	95.0	95.7 (93.2–98.3)
Cocaine	20/401	5.0	4.3 (1.7–6.8)
Frequency of injecting drugs in the past month			
Once per month	9/402	2.2	3.0 (0.6–5.4)
Few times per month	68/402	16.9	23.1 (15.7–30.4)
Once per week	9/402	2.2	1.5 (0.4–2.6)
Several times per week	91/402	22.6	27.0 (20.7–33.4)
Once per day	27/402	6.7	5.1 (2.8–7.3)
Several times per day	198/402	49.3	40.3 (33.3–47.3)
Ever shared needles or syringes with someone else during drug injection	260/402	64.7	60.4 (53.9–66.8)
Shared needles or syringes with someone else in the past month	57/399	14.3	14.1 (9.7–18.4)
Shared needles or syringes with someone else in the last episode of injection	31/401	7.7	8.4 (4.9–12.0)
Received drug dependence treatment^e^			
Currently	25/399	6.3	6.1 (3.3–8.9)
Not currently, but in the past	160/399	39.8	37.1 (31.3–42.9)
Never	214/399	53.4	56.7 (50.6–62.8)

^a^Unweighted estimates.

^b^Weighted estimates; RDS = respondent-driven sampling.

^c^95% CI = 95% confidence interval.

^d^Refers to current or most recent regular partner.

^e^Drug dependence treatment included opioid substitution therapy (as maintenance treatment), inpatient detoxification, outpatient drug dependence treatment, peer-based support groups.

A substantial number (68.9%) reported more than one sexual partner in the past 12 months while more than a third of PWID had four or more sexual partners in that time period.

Almost a half of PWID had experience of commercial sex, either in terms of paying or being paid for sex or exchanging sex for drugs, and 79.5% of these reported using a condom during the last commercial sexual intercourse.

A majority of participants (84.6%) reported using drugs sometimes or often before sexual intercourse in the past 12 months.

### Drug use behaviors

The median age of the first injecting drug use was 24 years (IQR = 20–28), while the median duration of injecting drugs was 5 years (IQR = 2–9). The vast majority of PWID reported using heroin (95.7%) followed by cocaine (4.3%), and no one reported using more than one type of a drug in a month preceding the survey (Table 1).

Injecting drugs at least once per day in a month before a survey was reported by 45.4% of PWID. Approximately two out of five respondents reported never sharing needles and syringes while injecting drugs. Sharing injecting equipment at least once during a month preceding the survey was reported by 14.1% of PWID while 8.4% shared injecting equipment last time they injected.

All but one respondent reported that they could obtain sterile injecting equipment when they needed it, either free-of-charge from harm reduction services or by buying them at pharmacies.

As sources of free-of-charge needles and syringes in the past 12 months respondents mentioned mobile outreach teams (8.0%), primary health-care centers (17.9%), and drop-in centers (53.5%).

A substantial proportion of respondents (60.8%) reported that they bought needles and syringes at pharmacies in the past 12 months.

During the past 12 months, 75.8% of PWID received risk-reduction counseling or some form of education about HIV prevention.

Table 2 shows the prevalence of needle and syringe sharing in the past month by socio-demographic and behavioral variables and results of bivariate and multivariate logistic regression.

**Table 2:** **Socio-demographic and behavioral correlates of sharing needles and syringes in the past month before a survey in Podgorica, Montenegro, 2013 (unweighted analysis)**

**Variable**	***n*** **/** ***N***	**Prevalence of sharing needles and syringes in the past month before a survey (%) with 95% CI**	**Unadjusted OR**	**Adjusted OR**
			**(95% CI)** ^**a**^	**(95% CI)**
Age group (years)			*p* = 0.054	*p* = 0.010^b^
≤25	4/64	6.2 (2.4–15.0)	1.00	1.00
≥26	53/335	15.8 (12.3–20.1)	2.81 (0.98–8.08)	4.16 (1.44–14.77)
Sex			*p* = 0.123	*p* = 0.039
Male	48/359	13.4 (10.2–17.3)	1.00	1.00
Female	9/40	22.5 (12.3–37.5)	1.88 (0.84–4.20)	2.53 (1.04–6.12)
Monthly income			*p* = 0.041	*p* = 0.051
>300 EUR	4/68	5.9 (2.3–14.2)	1.00	1.00
≤301 EUR	52/329	15.8 (12.3–20.1)	3.00 (1.05–8.60)	4.13 (0.99–18.77)
Frequency of injecting in the past month			*p* = 0.006	*p* = 0.008
Once per day or less	19/202	9.4 (6.1–14.2)	1.00	1.00
More than once per day	38/197	19.3 (14.4–25.4)	2.30 (1.27–4.15)	2.44 (1.25–4.73)
Number of sex partners in the past 12 months			*p* = 0.023	*p* = 0.009
≤3	26/242	10.7 (7.4–15.3)	1.00	1.00
≥4	28/147	19.1 (13.5–26.1)	1.95 (1.09–3.48)	2.35 (1.24–4.46)
Injected drugs in parks or on streets in the past month			*p* = 0.014	*p* = 0.010
No	41/330	12.4 (9.3–16.4)	1.00	1.00
Yes	16/66	24.2 (15.5–35.8)	2.25 (1.17–4.32)	2.61 (1.25–5.44)
Provided with free sterile needles and syringes in the past 12 months^c^			*p* = 0.009	*p* = 0.008
Yes	49/381	12.9 (9.9–16.6)	1.00	1.00
No	6/16	37.5 (18.5–61.4)	4.06 (1.41–11.68)	5.74 (1.58–20.9)

^a^OR = odds ratio; 95% CI = 95% confidence interval.

^b^ *p* values represent significance test for heterogeneity across the variable.

^c^This refers to being given free-of-charge needles and syringes by NGOs and/or the Primary Health Care Centre.

After adjustment in the multivariate analysis, significantly higher odds of needle and syringe sharing were found among PWID who were older than 26 years (aOR = 4.2, 95% CI 1.4–14.8), females, though only marginally higher (aOR = 2.5, 95% CI 1.0-6.1), those who injected drugs more frequently than once per day (aOR = 2.4, 95% CI 1.3–4.7), those who reported injecting drugs in parks or on streets in the past month (aOR = 2.6, 95% CI 1.3–5.4), those who reported not being provided with free-of-charge syringes and needles (aOR = 5.7, 95% CI 1.6–20.9), and those who had four and more sexual partners in the last 12 months (aOR = 2.4, 95% CI 1.2–4.5).

## Discussion

The HIV prevalence of 1.1% found in Podgorica in 2013 is higher than in the surveys done in 2008 and 2011, though this finding still implies a low-level HIV epidemic in PWID. The prevalence of HCV was at a similar level in 2013 (53.0%) as in 2008 and 2011.

Comparable HIV prevalence estimates were found in most recent surveys conducted in PWID in other countries of ex-Yugoslavia: 3% in Belgrade, Serbia in 2005; 0%–0.5% in several cities in Bosnia and Herzegovina in 2011; 0 in Croatia in 2007 and in FYR Macedonia in 2010 [12-15]. However, epidemics of HCV in PWID are well established in these countries—the prevalence in the abovementioned surveys was 66% in Belgrade, Serbia, 23%–49% in several cities in Bosnia and Herzegovina, 44%–65% in several cities in Croatia, and 70% in Skopje, FYR Macedonia [12-15].

It is encouraging that almost all respondents in this survey as in the 2008 and 2011 surveys (96.8% and 99.6%, respectively) reported that they could obtain sterile needles and syringes for injecting.

Sharing needles and syringes in a month prior to the survey was reported in 2013 by 14.1% of PWID, which is very similar to the findings from the survey carried out in 2011 (13.6%) and substantially less than in 2008 (24.2%), which might explain a stable prevalence of HCV in this time period.

However, there are several findings that show current gaps in HIV and HCV prevention and that have importance for further development of interventions for prevention of these infections in PWID in Montenegro.

Although a majority of PWID know where they can be tested for HIV, the HIV testing uptake is low—less than one in five respondents reported being tested for HIV in a year before the survey. A high proportion of respondents reported never being on drug dependence treatment. Interventions among PWID need to focus on ensuring better access to drug dependence treatment as it is highly effective in reducing injecting behaviors that put opioid-dependent injectors at risk for HIV and blood-borne infections [16,17].

Results of multivariate analysis highlighted that certain groups may benefit from stronger harm reduction programs that emphasize importance of consistent use of sterile injecting equipment, such as females who inject drugs (FWID), those who report higher number of sexual partners, and those who inject in outdoor settings.

It is well known that FWID are more difficult to reach with HIV prevention services compared to males and tend to engage in higher-risk injecting practices [18-21]. FWID usually depend on male partners for drugs and injections, leading to elevated drug and equipment sharing, and many engage in commercial sex. Given that FWID may be at higher risk of HIV, particularly via sexual transmission, there is a need to further explore barriers that they face in accessing harm reduction services. Separate prevalence assessment surveys can be done in FWID only to determine the level of HIV, HCV, and STIs in this sub-population and factors that expose them to higher risk of acquiring these infections.

There is evidence that PWID who engage in high-risk sexual behaviors might be more likely to engage in risky injection practices [22-25]. In our survey, almost a half of respondents reported ever selling or buying sex or exchanging it for drugs, and somewhat more than a half reported non-regular partners in the past 12 months and rather low condom use with such partners. Only a minority of respondents reported discussing HIV status before having the first intercourse with regular partners.

Of note is that in our study, older PWID reported significantly higher odds of needle and syringe sharing than younger PWID, which is a finding that has relevance for further intervention planning. Factors that put older PWID at risk of harmful injecting practices should be explored in Montenegro since research on the influence of age on the patterns of drug use mainly demonstrated that younger PWID are more likely than older PWID to share injecting equipment and engage in high-risk injecting behaviors [26-28].

Based on the results of this analysis, we recommend that free and confidential HIV testing and counseling should be more readily available for PWID as well as drug dependence treatment and sexual heath interventions that include screening for STIs, condom distribution programs, and promotion of safer sex practices. Since those who inject in outdoor settings had higher odds of needle and syringe sharing and a low proportion of PWID reported obtaining free-of-charge injecting equipment from outreach services, it is important to map sites in Podgorica where PWID gather so that harm reduction programs can be more effectively strengthened through outreach and mobile services.

This study was not without limitation. Self-reported behaviors are subject to social desirability and recall bias. Refusals to participate are challenging to measure in RDS and could have also biased the findings. RDS may have resulted in under-recruitment of certain types of participants, e.g., PWID with smaller injecting networks and women [29]. Better recruitment of females who inject drugs could have been achieved by having more female seeds. In the future HIV bio-behavioral surveys, efforts should be made to achieve more effective recruitment of FWID.

## Conclusion

The current evidence shows that the HIV epidemic in PWID in Montenegro is still at the low level, though the HIV prevalence might have increased in the period 2011–2013. The HCV epidemic is well established in this population as slightly more than a half of recruited PWID were found to be HCV infected. It is of paramount importance that targeted and effective interventions based on the combination of behavioral, biomedical, and structural approaches are kept sustainable and continue to be scaled up.

## Endnote

^a^Comprehensive knowledge of HIV meant knowing that proper and consistent use of condoms during sexual intercourse and having just one partner who is HIV negative and has no other partners can reduce the risk of getting HIV, knowing that a healthy-looking person can have HIV, knowing that it is possible to decrease the risk of HIV transmission by not sharing needles and other injecting equipment and rejecting the two most common local misconceptions about HIV transmission and prevention.
